# The CHEWMA Chart: A New Statistical Control Approach for Microclimate Monitoring in Preventive Conservation of Cultural Heritage

**DOI:** 10.3390/s25041242

**Published:** 2025-02-18

**Authors:** Ignacio Díaz-Arellano, Manuel Zarzo

**Affiliations:** Department of Applied Statistics and Operational Research and Quality, Universitat Politècnica de València, Camino de Vera s/n, 46022 Valencia, Spain; mazarcas@eio.upv.es

**Keywords:** preventive conservation, statistical process control, CHEWMA chart, microclimate monitoring, control chart, EN 15757:2010, cultural heritage

## Abstract

A new statistical control chart denoted as CHEWMA (Cultural Heritage EWMA) is proposed for microclimate monitoring in preventive conservation. This tool is a real-time detection method inspired by the EN 15757:2010 standard, serving as an alternative to its common adaptations. The proposed control chart is intended to detect short-term fluctuations (STFs) in temperature (T) and relative humidity (RH), which would enable timely interventions to mitigate the risk of mechanical damage to collections. The CHEWMA chart integrates the Exponentially Weighted Moving Average (EWMA) control chart with a weighting mechanism that prioritizes fluctuations occurring near extreme values. The methodology was validated using RH time series recorded by seven dataloggers installed at the Alava Fine Arts Museum, and, from these, seventy simulated time series were generated to enhance the robustness of the analyses. Sensitivity analyses demonstrated that, for the studied dataset, the CHEWMA chart exhibits stronger similarity to the application of EN 15757:2010 than other commonly used real-time STF detection methods in the literature. Furthermore, it provides a flexible option for real-time applications, enabling adaptation to specific conservation needs while remaining aligned with the general framework established by the standard. To the best of our knowledge, this is the first statistical process control chart designed for the field of preventive conservation of cultural heritage. Beyond assessing CHEWMA’s performance, this study reveals that, when adapting the procedures of the European norm by developing a new real-time approach based on a simple moving average (herein termed SMA-FT), a window of approximately 14 days is more appropriate for STF detection than the commonly assumed 30-day period in the literature.

## 1. Introduction

Temperature (T) and relative humidity (RH) are key factors in the deterioration of cultural heritage artifacts [1,2]. Therefore, the monitoring and assessment of indoor air conditions are essential for the preventive conservation of artifacts housed in cultural heritage institutions, such as museums, historical buildings, art galleries, and archives.

### 1.1. Norms and Guidelines: From Universal Constants to Acclimatization Perspective

Since the 1950s, the standards, regulations, and guidelines for the management of microclimatic conditions aiming to prevent the deterioration of cultural heritage have undergone significant changes [3]. In recent decades, it has been recognized that there are no universally ideal microclimatic conditions for the conservation of cultural heritage. In fact, maintaining artifacts under inadequate environmental conditions can damage them by not considering the microclimatic history to which the artifacts have adapted [4]. Furthermore, this rigid approach (e.g., strictly maintaining low temperatures at specific levels that are considered to be optimal for conservation) can cause discomfort for visitors [5,6] and an excessive increase in energy consumption in HVAC (heating, ventilation, and air conditioning) systems, thereby leading to higher economic and environmental costs.

As a result of these reported studies, a new generation of more flexible guidelines and standards have been proposed. Among them are the UNI 10969:2002 standard [7] and the EN 15757:2010 standard [8]. Additionally, the ASHRAE guidelines [9], although not a standardization body, provide standards and technical guidelines as an international professional society. These standards and guidelines take into account the previous conditions to which heritage artifacts have been exposed in order to establish appropriate microclimatic conditions. Among them, EN 15757:2010 prioritizes historical climate over other criteria and introduces the concept of acclimatization. This approach not only benefits the conservation of cultural heritage but also reduces energy demands. This historical evolution of the standards and guidelines is summarized in the review by Lucchi [10].

According to these standards, the diagnosis and prevention of short-term fluctuations (STFs) is especially important. Such a variability pattern is defined as a significant shift in RH or T, whether abrupt or accumulated over days, that can be potentially harmful to the mechanical properties of materials, particularly hygroscopic ones, generating cracks or permanent deformations [11]. The sensitivity to this response depends on various criteria, often conflicting with each other. Some of these criteria are the material of which the artifact is composed (e.g., paper [12] or wood [13]), the geometry [11], or previously received treatments [3,14]. Artifacts composed of different materials are particularly sensitive to STFs due to material interactions [15], while non-hygroscopic materials, such as ceramics or stone, can suffer from thermal shocks [16]. These scenarios are addressed in various standards, as reviewed by Guo et al. [17].

Given the difficulty in establishing the appropriate microclimatic conditions, multiple researchers have conducted risk assessments in real-world contexts using these norms and guidelines. For example, several studies are based on the EN 15757:2010 standard [18,19,20,21,22], UNI 10829:1999 [23,24], or ASHRAE guidelines [25]. Other researchers have focused on comparing their performance [26,27,28].

### 1.2. Real-Time Microclimatic Monitoring and Control Strategies

However, although adequate microclimatic conditions for the conservation of cultural heritage have been widely explored, the detection and real-time control of adverse conditions have been less extensively studied. The objective of microclimatic control strategies is to detect unfavorable conditions in real time and to intervene in order to apply corrective measures aimed at reducing the risk of damage. For this purpose, control strategies are based on methods designed for the detection of such conditions. These methods can be classified into two groups according to their approach: material-specific methods, which are based on models of the behavior of specific materials, and multi-material methods, which take into account the diversity of collections.

Material-specific methods use physical models to characterize the behavior of materials. However, they are limited to a narrow range of materials. Examples of this approach include the studies by Schellen and Van Schijndel [29], who establish a setpoint based on mechanical stress in wooden objects, and the research by Dionisi-Vici and Romano [30], which uses Equilibrium Moisture Content (EMC) as a basis to minimize the effects of climate on deformation and stress in wooden objects.

Multi-material methods can be divided into two subgroups: those based on existing standards or guidelines and those that propose alternative methods. The first subgroup comprises methods based on ASHRAE guidelines [31,32,33], while others are inspired by the EN 15757:2010 standard [34]. These adaptations benefit from the use of widely studied criteria. However, adapting a standard to create a new online detection method sometimes requires changes; otherwise, it might not be suitable for real-time monitoring or could be less effective as it is sometimes designed exclusively for offline risk assessment, based solely on preventive conservation criteria, focusing primarily on preventive conservation criteria sometimes at the expense of practical needs, or because a specific solution may address certain needs that a general framework may not fully cover. Additionally, the use of setpoints for microclimatic variables is a common practice that has been explored in several studies [35,36,37,38], aligning with stricter normative approaches. The second subgroup comprises methods that employ alternative criteria, for example, measuring the difference between outdoor and indoor humidity at the building where artifacts are located and using this information to activate a fan that dehumidifies the indoor air of a room [39,40,41]. Other approaches rely on statistical criteria, such as detecting STFs through outlier identification in differentiated time series of RH [42], or are based on risk assessment indices [36].

Some of these strategies have been compared in the literature. Broström et al. [43] compare setpoint strategies using an adapted version of the EN 15757:2010 norm, referred to as Natural Climate Fluctuations Control (NCfC). Other studies [44,45,46] evaluate a broad range of setpoint strategies, including dynamic setpoints based on or inspired by the ASHRAE climate classes. Finally, Coelho et al. [47] compare Thomson (Class 1) [48], ASHRAE (Class B), and FCT-UNL (Class 1) [49].

A review of the existing methods for real-time detection of conditions that present risks to the mechanical integrity of artifacts leads to the following conclusions: (i) statistical procedures are applied in a limited manner, although their proven success is in the monitoring and control of real-time processes in different fields; and (ii) there are few methods inspired by the EN 15757:2010 standard despite studies demonstrating the reliability and adaptability of this approach to different scenarios [27,50], along with its demonstrated low energy consumption [43,50]. This is particularly important because energy consumption in cultural heritage is expected to increase in the future due to climate change [51,52]. These remarks can be regarded as limitations of the state of the art.

### 1.3. Adapting Statistical Process Control Charts for Microclimatic Monitoring and Control

In order to address these limitations, the present work introduces CHEWMA, a novel detection method combining statistical process control (SPC) charts with the principles of the EN 15757:2010 standard. Firstly, these SPC charts, widely used in the statistical control of industrial processes, enable the detection of anomalous patterns in real time. Secondly, the method takes advantage of the extensively validated principles of the European standard, building on its proven strengths demonstrated in the scientific literature. It should be noted that the CHEWMA chart is not an alternative to the EN15757:2010 standard but rather a real-time adaptation that leverages its core principles of “historic climate” and “acclimatization”.

The objectives of the present study are the following: (i) to establish a methodology that is capable of adapting process control procedures to the microclimatic case for preventive conservation so that SPC charts can be applied in the field of preventive conservation; (ii) to develop a specific SPC chart based on this methodology that is specifically designed for materials that are sensitive to STFs; and (iii) to discuss the advantages and limitations of the most commonly used real-time STF detection methods in the literature and evaluate the proposed control chart in comparison with them.

The present paper is organized into five main sections: (i) Methodological Framework, which conducts a comprehensive review on the adaptation of statistical process control to non-industrial fields, as well as how to carry out this adaptation in the microclimatic context, and, as a result, the proposed methodology focused on the CHEWMA chart; (ii) Materials and Methods, which detail the monitoring campaign carried out, the procedure for generating simulated data, and the methods applied to evaluate the proposed approach; (iii) Results and Discussion, where the sensitivity of the CHEWMA chart parameters is analyzed and compared to similar methods reported in the literature; (iv) Conclusions, summarizing the main findings; and (v), Future Work, which outlines the hypotheses to be addressed in future studies and potential research opportunities.

## 2. Methodological Framework

### 2.1. Control Charts for Process Monitoring

In the 1920s, WA Shewhart laid the foundational principles still employed today in the design and implementation of SPC charts [53]. Their objective is to distinguish random variability (i.e., caused by unknown or uncontrollable but estimable factors) from assignable causes affecting the process (e.g., breakage or wear of a machine part in the industrial case). This random variability is assessed when the process is assumed to be under control (i.e., operating as expected). The estimated variability is used to construct the control chart, in a stage referred to as Phase 1. Once the chart is operational, a sample of the variable to be monitored is collected periodically. This stage is known as Phase 2.

In the classical approach to SPC charts, upper and lower limits are typically set to establish the range of values within which the random variations in the process are expected to occur under statistical control conditions (i.e., when no assignable causes are altering the inherent variability regarding the process). From each sample, the statistic to be monitored (e.g., the sample mean) is computed and plotted on the chart. When the value of this statistic falls within the established limits, no action is required, indicating that the process remains under control. However, when it falls outside the limits, it is referred to as a signal or an out-of-control signal, which can be interpreted in two ways: either the process has been correctly diagnosed as out of control (i.e., the statistical variability pattern of the monitored statistic has changed) or, conversely, the process remains under control but a Type I error has occurred (i.e., a false positive). Similarly, if the process is out of statistical control but remains undetected by the chart, a Type II error has occurred (i.e., a false negative). When an out-of-control signal is detected, it is generally assumed that the expected variability pattern of the monitored statistic has been altered. From that point forward, it is necessary to implement the measures outlined in a protocol to investigate the cause and restore the process to be under statistical control.

### 2.2. Statistical Control Charts Applied to Non-Industrial Fields

SPC charts have been successfully used for the monitoring of processes and to detect anomalous patterns, and their application has been extended beyond industrial contexts. Applying this tool to non-industrial areas such as microclimatic monitoring involves methodological differences, requiring a review of the limitations and adaptations that researchers have encountered when implementing SPC charts in their respective fields.

One of the areas where their application has been most extensively expanded is the healthcare sector. Several studies [54,55] emphasize the importance of analyzing and adapting to the specific characteristics of the healthcare sector compared to industrial environments, highlighting differences in data nature, sampling frequency, and the challenges of collecting controlled samples for chart construction. They also note that these differences should be considered when evaluating the adaptation of SPC methodologies. Speroff and O’Connor [56] emphasize the common presence of autocorrelation, which has been observed in various cases, such as optimizing the use of inhaled corticosteroids or adjusting environmental controls to enhance the peak expiratory flow rates in asthmatic patients. Thor et al. [57] also emphasize the presence of autocorrelation and highlight the need to assess the limitations of SPC charts in the medical context and accordingly adapt the SPC methodology. Among these adaptations is the need for data preprocessing, also discussed by Koetsier et al. [58]. Finally, Suman and Prajapati [59] highlight the difficulty of obtaining adequate data to adjust the chart’s parameters in Phase I.

Regarding the application of SPC charts in natural resource management, Mertens et al. [60] point out that autocorrelation is a common problem and that a controlled sample is usually obtained through preprocessing steps such as outlier elimination. These authors also indicate, like Krieter et al. [61], that the EWMA chart, which is a type of “memory” SPC chart, is convenient due to its ease of understanding. Petitgas [62] utilizes data from a period deemed adequate as a sample under control.

With respect to the quality control in wastewater treatment plants, SPC charts have also been employed [63,64]. In particular, Smeti et al. [63] address autocorrelation by monitoring and controlling the residuals of an ARIMA model. Regarding environmental management, Morrison [65] proposes to determine the position of the chart’s central line based on what is “subjectively” considered adequate for the process given the difficulty in obtaining a sample statistically under control in this area, such as when controlling the population of long-lived organisms. Gove et al. [66] adopt a similar approach by establishing control limits based on the analysis of empirical models and expert knowledge. Regarding SPC charts in forest-fire management, Podur et al. [67] reiterate the difficulty of obtaining adequate data as a drawback.

In climatological studies, Villeta et al. [68] integrate autocorrelation through Markov chains to detect days with extreme temperatures, Sengupta et al. [69] utilize this approach as an analytical tool, and Rashid et al. [70] apply an EWMA chart for the early detection of flood hazards.

SPC charts have also been applied in financial management, where Kovářík et al. [71] emphasize the need to adequately manage autocorrelation and evaluate the methodology by analyzing various case studies. In text stream analysis, Ashton et al. [72] monitor consumer satisfaction based on their comments. For this purpose, these authors first apply a support vector machine (SVM), the results of which are monitored using an SPC chart, enhancing the interpretability of the outcomes compared to other systems.

Two key conclusions regarding the challenges and corresponding adaptations discussed have been identified: (i) it becomes difficult to obtain a sample statistically under control, which is required to establish the chart’s parameters in Phase I, because the variables being monitored are not derived from a “controlled” process. Therefore, data are typically preprocessed, and expert criteria are used to determine the control chart’s parameters. (ii) When addressing the challenge of autocorrelation, which is very common in these applications, one of three strategies is generally adopted: (a) empirically adjusting control limits based on the desired frequency of out-of-limit values; (b) integrating autocorrelation into the SPC chart as part of the statistical modeling, for example, by incorporating autocorrelation into Markov chains [68]; or (c) modeling autocorrelation with time series models, which enables the monitoring of residuals, defined as the differences between observed data and predictions from a time series model.

### 2.3. Statistical Control Charts Applied to Microclimates

The deployment of SPC charts for microclimate monitoring in the context of cultural heritage, particularly for controlling the dynamics of RH and T, also requires designing a specific methodology to adapt the statistical properties commonly considered in the field of process control. For this purpose, the key differences between SPC in industrial and microclimatic contexts must first be identified, many of which have already been addressed in the aforementioned applications in non-industrial fields.

The first distinctive characteristic is the lack of control over the factors that cause variability among thermohygrometric variables. While industrial processes often benefit from a better understanding of which factors affect the variability of a given parameter, enabling the modeling of its temporal dynamics as long as these factors remain unchanged, the capability to model the temporal dynamics of variables such as RH or T is usually much more limited. Although some cultural heritage institutions have HVAC systems, their impact on the variability among RH and T is limited. Moreover, the impact of these systems is preferable to be as minimal as possible in order to reduce energy consumption and decrease the likelihood of improper use that might cause mechanical damage to artifacts. In the case of microclimates, most of the variability is caused by external climatic conditions uncontrollable by cultural heritage institutions, although they can be mitigated with passive measures (e.g., architectural design, construction materials, etc.) and reactive measures (e.g., HVAC systems, opening windows or doors to generate air currents, etc.). However, there may also be causal factors controllable by the institution itself (e.g., excess of people in the venue, inappropriate ventilation circuits, or the aforementioned HVAC systems).

This lack of control over causal factors has multiple consequences. Firstly, it is difficult to define a process as being under statistical control if, under normal operating conditions (i.e., not being adversely affected by any controllable factors), the thermohygrometric conditions remain unpredictable from the cultural heritage institution’s perspective. Therefore, in order to estimate the appropriate parameters for the control chart, it is necessary to rely on the distribution of the idealized time series, simulating conditions under control through adequate data preprocessing. The second consequence is that assigning causes to out-of-control signals becomes a more diffuse task than in the industrial context. When an out-of-control signal is detected and corrective measures need to be taken, possible causal factors must be identified, which may or may not be under the cultural heritage institution’s control. Consequently, the options are either to correct them by directly addressing the cause or to activate reactive measures that mitigate or counteract the undesired situation.

In microclimatic monitoring, a recording frequency of T/RH less than 1 h is recommended to avoid losing relevant characteristics of microclimate dynamics. As a result, observations become highly autocorrelated, both consecutively and seasonally. However, classical SPC charts assume the independence of observations, which implies the absence of autocorrelation. Despite the relevance and increasing occurrence of autocorrelation with the advancement in sensorization in multiple fields, it is common to assume ideal conditions and violate this assumption [73]. Section 2.7 discusses how to manage autocorrelation in the microclimatic context.

On the other hand, in order to establish the parameters of a control chart (e.g., control limits, sampling frequency, etc.), it is necessary to consider economic and engineering criteria, taking into account the statistical properties of the charts. The economic factors to consider are primarily the cost of sampling, losses associated with defective production, and the investigation of out-of-control signals, which are actually false alarms [74]. In [75], the adjustment of the chart’s parameters is proposed based on the optimization of a cost function that considers the aforementioned variables. Other factors involve indirect and hardly quantifiable costs, such as the distrust and disuse of the tool by the staff due to an excessive number of Type I errors resulting from the chart.

In the microclimatic case, the costs of implementing a statistical control system present particularities that must be taken into account. First, since microclimate monitoring is carried out through sensors, the sampling frequency can generally be relatively high as it does not involve significant extra costs when such monitoring is performed in real time wirelessly or with wired sensors [76]. Second, estimating whether an out-of-control signal is a genuine alarm or a Type I error makes little practical sense because it is assumed that there is no control over some of the factors responsible for microclimate variability, in this case RH and T. Therefore, once an out-of-control signal is detected, it simply indicates that extreme conditions, which may need to be corrected or mitigated, have been identified. When such signals are detected, there are no costs associated with stopping the production, nor does it lead to technicians distrusting the tool given that Type I errors cannot occur. Third, no defective pieces or their associated problems are produced, but there are potential economic and patrimonial costs of damaging a heritage artifact. These potential costs must be compared against the cost associated with having many out-of-control signals and, therefore, the cultural heritage institution’s staff and systems reacting to them.

In conclusion, in the particular case of SPC charts applied to microclimate control, there are many similarities with other applications in non-industrial fields. We can determine that these differences are the following: (i) in practice, obtaining an annual sample “under statistical control” is not feasible, making data preprocessing and expert criteria essential to derive a controlled sample or to identify which microclimatic dynamics are acceptable or problematic; (ii) in many cases, there is no direct control over the main causal factors driving the process to be out of statistical control. As a result, the regulation of microclimatic conditions often requires mitigating or counteracting the effect of those factors; (iii) in general, the sampling frequency can be relatively high, leading to time series with a strong positive autocorrelation; and (iv) the costs associated with microclimate monitoring focus on the potential damage to artifacts and the cost of staff reacting to out-of-control signals.

### 2.4. EWMA Control Chart

The SPC chart known as EWMA (Exponentially Weighted Moving Average) [77] is a “memory” control chart, meaning that it takes into account past observations to monitor the state of the process at a given moment. In this chart, it is customary for the sample size to be one, and therefore the samples are referred to as observations [78]. The statistic plotted on this chart, *Z*, is computed by averaging the observations in a weighted manner through a geometrically decreasing progression.

In this manner, the most recently recorded observation receives the highest weight, the previous one less, and so forth, as indicated in Equation (1), where $X_{t}$ represents the monitored variable at time point *t* and $Z_{t}$ the calculated statistic for that observation. This weighting uses the constant λ, whose value ranges between zero (exclusive) and one (inclusive).

(1) $$Z_{t}=\lambda X_{t}+\left(1-\lambda \right)Z_{t-1},\;t\in N,\lambda \in \left(0,1\right]$$

If the independence of observations is assumed, the variance can be calculated as follows for each instant *t* [79]:

(2) $$\sigma_{Z_{t}}^{2}=\sigma_{X}^{2}\left(\frac{\lambda }{2-\lambda }\right)\left[1-{\left(1-\lambda \right)}^{2t}\right]$$

As *t* becomes increasingly large, the previous equation can be approximated as

(3) $$\sigma_{Z}^{2}=\sigma_{X}^{2}\left(\frac{\lambda }{2-\lambda }\right)$$

One STF can be defined as an abrupt variation in T or RH over time, being the magnitude of the change relative to the time over which it occurs. This rate of change can be assessed by differentiating the series (i.e., subtracting each observation from the previous one, thereby obtaining the increments and decrements of T or RH). In the detection method proposed by Tringa et al. [42], this procedure is followed, and an STF is identified when one of the hourly increments or decrements recorded is extreme (i.e., significantly deviating from the mean of the set of increments/decrements). Hence, an STF would correspond to a significant shift between consecutive records.

However, while an abrupt STF lasting minutes or an hour can indeed affect some materials like paper [12], STFs that impact the mechanical properties of many other materials occur over hours or days (e.g., wood [13]). These STFs cannot be detected as alert signals unless their accumulation over time is taken into account, regardless of the sampling frequency. For example, a small 0.5% RH increase every hour over a 24-h period may result in a significant and hazardous STF for many materials, yet it could remain unnoticed if only the magnitude of individual increments and decrements is considered separately. A statistical control strategy intended to be used across different collections must be sufficiently flexible to account for the diverse behavior of the materials. The “memory” weighting of the EWMA chart appears suitable for the detection of these STFs as each $Z_{t}$ represents the accumulation of past increments and decrements, providing more weight to recent observations.

In the present study, our approach is based on the EWMA chart due to its simplicity, versatility, and suitability for detecting STFs. Other approaches, such as the CUSUM chart [80], require specifying a certain minimum shift to consider any signal as out of control, which can be too restrictive for the detection of STFs in collections of multiple materials. Moreover, it is a more challenging control chart to interpret and apply, particularly for individuals who are unfamiliar with statistical process control.

### 2.5. CHEWMA: EWMA Control Chart Adapted to Preventive Conservation of Cultural Heritage

The risk of STFs to the mechanical properties of materials depends on the magnitude of the change relative to the time over which it occurs. However, this risk increases when the STF approaches extreme values. For example, a 10% RH change is generally more hazardous if it occurs over 24 h rather than 10 days. Additionally, the same RH change is typically more dangerous when it occurs at extreme levels (e.g., from 20% to 10% RH) than at central values (e.g., from 45% to 55% RH). This issue applies to multiple materials like wood [11,81,82], silk [83], ceramics or artifacts with a layered structure (e.g., shells or skull bones) [16], biological specimens [84,85], concrete [86], and it is particularly critical in artifacts composed of composite materials [16]. A key study that contributed to the development of the EN 15757:2010 standard [87] incorporates this increased risk into its methodology for identifying STFs.

Aimed at taking into account this risk in the EWMA chart, we propose an adaptation called Cultural Heritage EWMA (CHEWMA) control chart. This chart is a modification of EWMA where not only the weight of past observations is considered but also each observation is linearly weighted based on its distance from a reference point *p* established by the user (e.g., 50% RH). In this manner, STFs occurring near extreme values (e.g., near 0% or 100% RH) are considered potentially more hazardous.

In order to use this CHEWMA chart, the process begins with the observed time series *X* of either RH or T measurements. First, the differentiated series ΔX is calculated, representing the increments or decrements of RH or T at each time point *t*, as follows:

(4) $$\Delta X_{t}=X_{t}-X_{t-1}$$

Secondly, a simple moving average (SMA) is calculated from observations of the last 24-h period, denoted as $X_{t}^{24h}$. It is computed as the following average:

(5) $$X_{t}^{24h}=\frac{1}{|I_{t}|}\sum_{j\in I_{t}}X_{j},\;I_{t}=\left\{t-23,t-22,\ldots ,t\right\},$$

where $|I_{t}|$ is the number of observations (*X*) available in the last 24 h for the time point *t*. The specific set of time points $I_{t}$ has been defined assuming an hourly recording frequency as a common example, although it could be any other.

With these two variables, $\Delta X_{t}$ and $X_{t}^{24h}$, the weighted variable *W* is calculated for each time point *t* as follows:

(6) $$W_{t}=\Delta X_{t}+\beta \left(\frac{X_{t}^{24h}}{p}-1\right),\beta \geq 0,p>0.$$

In this manner, an addition term is applied to the increment or decrement $\Delta X_{t}$ based on the deviation of $X_{t}^{24h}$ from the reference point *p*, which is multiplied by a weighting factor β. For example, if the 24-h moving average of RH is 70% (i.e., $X_{t}^{24h}=70$) and the reference RH is 50% (i.e., p=50), a positive addition term of 0.4 would be applied (i.e., (70/50)−1=0.4). Therefore, β regulates the magnitude of this adjustment, making it larger (β>1), smaller or equal (β≤1), or even null (β=0). Conversely, if $X_{t}^{24h}=50$, no addition term would be applied (i.e., (50/50)−1=0) as the daily moving average coincides with the reference point.

The aforementioned statistic $W_{t}$ is subsequently used to calculate the EWMA-specific statistic $Z_{t}$ which, following Equation (1), is calculated as

(7) $$Z_{t}=\lambda W_{t}+\left(1-\lambda \right)Z_{i-t}$$

Based on this formulation and assuming ΔX and $X_{t}^{24h}$ to be Gaussian and independent, and given that $\mu_{\Delta X}=0$, it turns out (see Appendix A) that

(8) $${\hat{\mu }}_{Z_{t}}={\hat{\mu }}_{W_{t}}=-\beta +\frac{\beta }{p}{\hat{\mu }}_{X_{t}^{24h}}$$

(9) $${\hat{\sigma }}_{W_{t}}^{2}={\hat{\sigma }}_{\Delta X}^{2}+{\left(\frac{\beta }{p}\right)}^{2}{\hat{\sigma }}_{X_{t}^{24h}}^{2}$$

From Equation (2), it follows that

(10) $${\hat{\sigma }}_{Z_{t}}^{2}={\hat{\sigma }}_{W_{t}}^{2}\left(\frac{\lambda }{2-\lambda }\right)\left[1-{\left(1-\lambda \right)}^{2t}\right]$$

Note that, when β=0, the addition term is nullified, and the chart becomes a standard EWMA chart applied to the differenced series. In this case, only Equation (7) would be applied to the differenced time series. This configuration should be used when STFs are not considered significantly more hazardous at extreme absolute values (e.g., 10% or 90% RH).

When λ=1 is chosen in addition to setting β=0, the chart becomes a Shewhart chart, resulting in a configuration similar to the fluctuation detection method proposed by Tringa et al. [42]. In this sense, the CHEWMA chart can be regarded as a generalization. This configuration (i.e., β=0, λ=1) should be used when the goal is to detect very abrupt STFs occurring at a rate equal to the recording period (e.g., for artifacts composed of materials that respond quickly to shifts in RH or T). As β increases, the addition term that penalizes extreme observations also becomes more pronounced. For λ=1 and β>0, the chart becomes a Shewhart chart with weighted observations. Conversely, λ should be reduced when the chart is adjusted to detect longer fluctuations (e.g., for artifacts composed of materials with a slow response to shifts in RH or T).

The statistics required for the SPC chart’s operation are $\mu_{X_{t}^{24h}}$, $\sigma_{X_{t}^{24h}}$, and $\sigma_{\Delta X}$. The constants to be established are λ, β, *p*, and *L*. The selection of λ, as well as the reference point *p*, the factor β for linearly weighting observations, and the distance *L* for the adjustment of the control limits (addressed below in Section 2.6.2) depends on the specific needs of the cultural heritage institution. These needs include preventive conservation requirements (i.e., the materials’ properties, their acclimatization to past conditions, their conservation state, etc.) and economic considerations (e.g., the amount of out-of-control signals that can be managed, or the potential cost of artifact deterioration).

### 2.6. Construction and Implementation of the CHEWMA Control Chart

**Figure 1:**
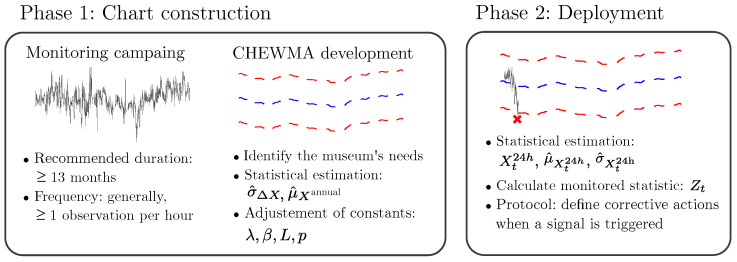
Summary of the phases for constructing and deploying the CHEWMA chart based on classical SPC methodology. First, a monitoring campaign is conducted, followed by the statistical estimation and adjustment of the constants. The chart is then validated using historical data to ensure that it generates the desired amount of alarm signals aligned with the cultural heritage institution’s needs. During the deployment phase, the necessary statistics for calculating the control limits are estimated, and the monitoring statistic *Z* is computed. In the event of a signal, the established protocol is triggered to mitigate adverse conditions. Periodic validation is essential to ensure that the process remains stable and the chart continues to be appropriate for microclimate monitoring.

Once the CHEWMA chart has been introduced, this section outlines the proposed methodology for applying this chart to microclimatic scenarios in order to detect STFs. The fundamental steps for the implementation of this chart are illustrated in Figure 1.

#### 2.6.1. Phase 1: Construction of the Control Chart

The control chart is constructed during Phase 1. In this stage, the statistics ${\hat{\sigma }}_{\Delta X}$ and ${\hat{\mu }}_{X^{\mathrm{annual}}}$ are computed. Moreover, the constants λ, β, *p*, and *L* are adjusted (the specific role of ${\hat{\mu }}_{X^{\mathrm{annual}}}$ will be detailed in Section 2.6.2). This adjustment involves applying the CHEWMA chart to historical time series data and evaluating the number and type of STFs detected under different configurations. This methodology aligns with the approaches proposed in [65,66], who suggested setting control limits based on empirical model analysis and expert knowledge when adapting control charts to their respective fields.

When using historical data, it is essential to ensure that no significantly abnormal patterns are observed (e.g., no major failures occurred in HVAC systems). If this condition is fulfilled, data from a single year can reflect the temporal dynamics of the most relevant seasonal components [76]. However, if data spanning multiple years are available, it is recommended to use them for a more accurate characterization of RH and T temporal dynamics. If observations for an entire year are not yet available, the SPC chart can still be configured using data from fewer months, although biases may be introduced. The minimum number of months required depends on prior knowledge of the microclimate (e.g., whether it is relatively stable or not) and the risks being addressed.

In order to calculate ${\hat{\sigma }}_{\Delta X}$, the differentiated series of *X* is computed, outliers are removed, and the standard deviation is computed. In the present study, those values exceeding three standard deviations were regarded as outliers and removed, although alternative methods for outlier removal may also be suitable. As discussed in Section 2.3, it is not possible to obtain a sample statistically under control in the same manner as in classical SPC chart applications. Therefore, preprocessing steps, such as removing outliers, are necessary to obtain a more accurate estimation of the required statistics.

While $X_{t}^{24h}$ is calculated as the SMA of the observations recorded over the last 24 h with respect to time point *t*, ${\hat{\mu }}_{X_{t}^{24h}}$ is computed as the SMA of the observations over the past 14 days relative to the same time point. To calculate ${\hat{\sigma }}_{X_{t}^{24h}}$, the observed series *X* is first deseasonalized by subtracting ${\hat{\mu }}_{X_{t}^{24h}}$, which leads to the deseasonalized series. Then, for each time point *t* in the deseasonalized series, the set of values from the previous 14 days is considered, outliers are removed, and the standard deviation is computed. A 14-day window was chosen because it provides a suitable balance between accurate estimations and the performance of the CHEWMA chart according to different tests carried out. This procedure aligns with approaches used by SPC charts with moving windows (e.g., [88]), which estimate SPC chart statistics using observations within a specific moving window.

It is important to note that ${\hat{\sigma }}_{\Delta X}$ and ${\hat{\mu }}_{X^{\mathrm{annual}}}$ are estimated from historical records of previous years and are therefore only calculated in Phase 1. By contrast, the statistics ${\hat{\mu }}_{X_{t}^{24h}}$ and $\hat{\sigma }X_{t}^{24h}$ are computed in both stages: first in Phase 1, based on historical data, when the CHEWMA chart is applied to adjust the constants according to the cultural heritage institution’s needs; and second, in Phase 2, during the operational deployment of the chart.

**Figure 2:**
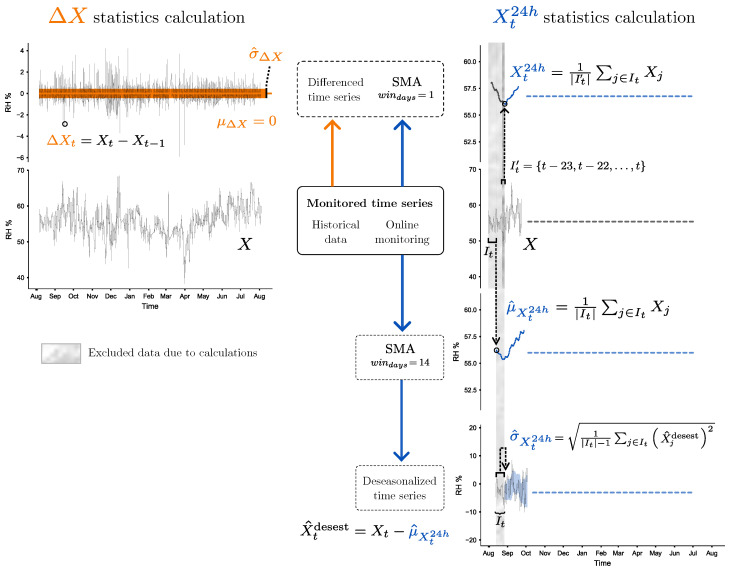
Steps for the calculation of the statistics derived from ΔX and $X_{t}^{24h}$ based on the recorded time series *X*. The estimation of ${\hat{\sigma }}_{\Delta X}$ is performed during Phase 1. On the other hand, ${\hat{\mu }}_{X_{t}^{24h}}$ and ${\hat{\sigma }}_{X_{t}^{24h}}$ are calculated upon the application of CHEWMA. Thus, these statistics are computed in Phase 2, during CHEWMA’s operational phase aimed at STF detection, and in Phase 1, when the SPC chart is applied to historical data to adjust constants λ, β, *p*, and *L*. The chart assumes that outliers from the differentiated time series (ΔX) and the deseasonalized series (${\hat{X}}_{t}^{\mathrm{desest}}$) have been removed. It is also assumed that the recording frequency of *X* is one observation per hour.

Although the calculation of ${\hat{\mu }}_{X_{t}^{24h}}$ and ${\hat{\sigma }}_{X_{t}^{24h}}$ requires a temporal window of 28 days (i.e., 14 days for the estimation of each statistic), the estimation of statistics with lower windows can be assumed until sufficient observations have been recorded. Hence, the CHEWMA chart becomes operational after 14 days of use. In Figure 2, the shaded region corresponds to excluded data used for these calculations. However, this time frame is only required when β>0. This additional complexity is required for the chart to account for the proximity of fluctuations to extreme values. When this is not a critical need for the preservation of the mechanical properties of the artifacts, it is recommended to set β=0. In this way, the control chart becomes simpler and only requires the calculation and estimation of the statistics of ΔX and can be deployed as soon as observations are available (see Table 1).

Since the thermohygrometric conditions to which collections are exposed may evolve over time, it is advisable to periodically update the chart parameters. First, as previously discussed, records spanning several years are recommended for estimating the chart statistics because observations from a single annual cycle may not be sufficiently representative. In the absence of multi-year time series, Litti and Audenaert [76] suggest performing updates annually. Furthermore, in the long term, it is important to consider potential shifts in climate [27], such as those driven by global climate change, whose impact on cultural heritage conservation is expected to vary depending on the specific climate zone [89]. However, when using records from periods during which microclimate interventions have been implemented, it is crucial to recognize that these records may no longer reflect the natural dynamics of the microclimate. This should be taken into account to avoid inadvertently imposing increasingly restrictive conditions with each successive update of the CHEWMA chart.

#### 2.6.2. Establishment of Control Limits

A particularly critical aspect of Phase 1 is the proper establishment of control limits. Similar to the limits calculated for a standard EWMA chart, the upper control limit (UCL) and lower control limit (LCL) of the CHEWMA chart are determined as follows:

(11) $${\mathrm{CL}}_{t}={\hat{\mu }}_{Z_{t}}^{\prime }\pm L{\hat{\sigma }}_{Z_{t}},$$

where *L* is a constant that enables the control limits to be widened or narrowed.

However, to effectively weight extreme fluctuations, the calculation of ${\hat{\mu }}_{Z_{t}}$ for ${\mathrm{CL}}_{t}$ must be adjusted by replacing the constant *p* with the estimated annual mean of *X*. This adjustment modifies the definitions of $\mu_{Z_{t}}$ and $\mu_{W_{t}}$ as follows:

(12) $${\hat{\mu }}_{Z_{t}}^{\prime }={\hat{\mu }}_{W_{t}}^{\prime }=-\beta +\beta \left(\frac{{\hat{\mu }}_{X_{t}^{24h}}}{{\hat{\mu }}_{X^{\mathrm{annual}}}}\right)$$

**Figure 3:**
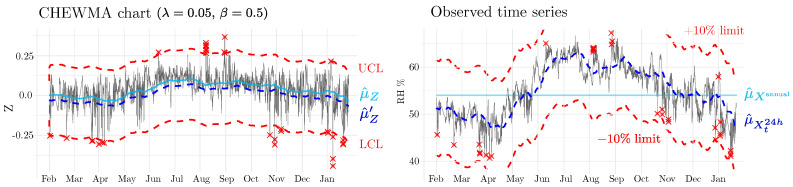
CHEWMA chart with λ=0.05 and β=0.5 on a real time series. As observed, since the annual mean of *X* is greater than the RH reference point (i.e., ${\hat{\mu }}_{X^{\mathrm{annual}}}>p$), it follows that ${\hat{\mu }}_{Z_{t}}^{\prime }<{\hat{\mu }}_{Z_{t}}$. Consequently, the UCL and LCL limits, centered around ${\hat{\mu }}_{Z_{t}}^{\prime }$, are positioned asymmetrically relative to the actual mean of the *Z* statistic (i.e., ${\hat{\mu }}_{Z_{t}}$). The $Z_{t}$ values that exceed the limits UCL and LCL are marked as signals in red. Optional limits corresponding to ±10% RH are added to the observed time series.

In this way, control limits are placed asymmetrically around $Z_{t}$ when RH or T approach extreme values. This adjustment enables the chart to become more sensitive to STFs at extreme values. According to Equation (12), as ${\hat{\mu }}_{X_{t}^{24h}}\neq {\hat{\mu }}_{X^{\mathrm{annual}}}$, the modified mean shifts from zero (Figure 3). In these circumstances, when calculating ${\hat{\mu }}_{Z_{t}}^{\prime }$ by replacing *p* with ${\hat{\mu }}_{X^{\mathrm{annual}}}$, the limits are positioned asymmetrically around the real mean of the monitored statistic *Z*. For example, if the annual mean of the observed series (i.e., ${\hat{\mu }}_{X^{\mathrm{annual}}}=60\%$) and the reference RH is 50% (i.e., p=50%), then ${\hat{\mu }}_{Z_{t}}^{\prime }<{\hat{\mu }}_{Z_{t}}$ and the upper limit will be closer to the observations than the lower limit. Therefore, it will be more likely for an STF to be detected by exceeding the upper limit.

Note that this behavior of the limits occurs when β≠0. Otherwise, ${\hat{\mu }}_{Z_{t}}^{\prime }=0$ for all time points *t*, and the limits are symmetrically positioned around the statistic *Z*.

Although ${\hat{\mu }}_{X^{\mathrm{annual}}}$ must be calculated during Phase 1 using historical data, it is a relatively straightforward statistic to estimate that can be computed with a relatively low recording frequency, although it does require the complete annual period of RH or T data.

The appropriate value of the parameter *L* is determined by applying the chart to the observed historical series and relying on the expertise of conservation specialists. Based on the value of *L* (Equation (11)), the control limits are either narrowed or widened, thereby defining the amount and frequency of unacceptable STFs for the context in which the control chart will operate.

The EN 15757:2010 standard states that, if RH fluctuations deviate by less than 10% from the seasonal RH level, a new 10% RH threshold may be set “under the responsibility of a qualified conservation professional” (i.e., if a conservation professional deems these conditions appropriate for the artifact). Hereafter, this will be referred to as the 10% criterion. In CHEWMA, this criterion is adapted from the standard’s guidelines by defining upper and lower limits around ${\hat{\mu }}_{X_{t}^{24h}}$, adding and subtracting 10%, respectively (Figure 3). From the perspective of SPC, these additional limits might be interpreted as tolerance limits.

#### 2.6.3. Phase 2: Operational Deployment of the Control Chart

**Figure 4:**
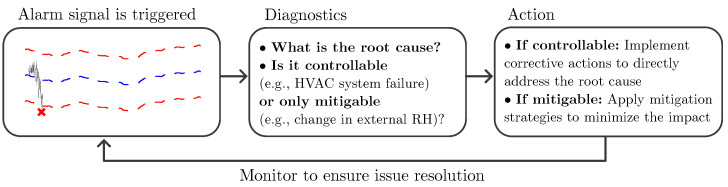
Main flowchart outlining the recommended steps to undertake when an alarm signal is triggered in the CHEWMA chart.

Once the construction of this CHEWMA chart is complete, its operational deployment begins. In order to start the process monitoring, the statistic $Z_{t}$ must be calculated for each new observation (as detailed in Section 2.5). When an alarm signal is triggered, a specific protocol must be followed in order to address the adverse situation. Figure 4 provides an overview of the protocol’s recommended steps.

Alarm signals are triggered at time point *t* when the value of $Z_{t}$ exceeds the upper control limit (${\mathrm{UCL}}_{t}$) or it falls below the lower control limit (${\mathrm{LCL}}_{t}$). Otherwise, no signal is generated.

Upon a signal, it is recommended to diagnose the root cause of the alarm and determine whether the STF is controllable (e.g., incorrect thermostat programming) or mitigable (e.g., extreme external weather conditions). Based on this classification, corrective actions should be applied to eliminate the cause, or mitigation measures should be implemented to reduce its impact. Although the mechanism most commonly described in the literature is the activation or deactivation of HVAC systems, other approaches comprise regulating the number of people in the venue [90] or even opening and closing windows [32,41]. The method of intervention is site-specific and depends on knowledge of the microclimate’s behavior and the local conditions. Following the intervention, attention should be paid to the control chart over the following hours to evaluate whether the detected STF has been effectively mitigated. This signal-diagnosis cycle enables a deeper understanding of the microclimate and the online use of the CHEWMA chart.

In industrial applications of control charts, once an out-of-control signal appears and the process has been subsequently corrected to bring it back under statistical control, it is common practice to reset the chart when past observations are weighted. This prevents Type I errors by restarting all statistical calculations and establishing the new observation as the initial reference point (t=1). This implies that averages, standard deviations, and other control chart statistics are recalculated using only the observations recorded from that point onward, discarding the impact of prior observations. However, in this application, the chart should not be reset due to the lack of control over causal factors and the high autocorrelation of observations, thus preventing the process from being considered under control after an intervention.

The application of the CHEWMA chart involves the visual inspection of two plots (see example in Figure 3): on the one hand, a chart monitoring the *Z* statistic, which displays the control limits (${\mathrm{UCL}}_{t}$ and ${\mathrm{LCL}}_{t}$) and the generated signals, and, simultaneously, another chart that displays the observed time series and the detected signals. The combination of both plots serves as a crucial tool for correctly interpreting the performance of this control chart, leading to a correct understanding of the evolution of the monitored variable, and thus making informed decisions.

For more sophisticated systems, interventions following a control chart signal can be automated by linking CHEWMA to HVAC regulation systems.

### 2.7. Control Charts for Autocorrelated Processes

The most widely studied control charts (e.g., the Shewhart, EWMA, and CUSUM chart) typically assume that the observations of the monitored variable are independent over time. However, as commented above, such an assumption is often violated in practice. This issue is particularly prominent in processes with inherent inertia (e.g., chemical processes) and in systems monitored automatically via sensorization, where high-frequency sampling is feasible [73]. Microclimatic monitoring exemplifies such scenarios, requiring an investigation into the potential impacts of autocorrelated measurements on SPC charts, such as the CHEWMA chart.

When positive autocorrelation in the case of microclimatic contexts is not taken into account, it results in underestimated control limits, which, in turn, lead to an increased rate of false alarms [91]. Memory-based charts in terms of EWMA and the CUSUM chart are particularly sensitive to this problem. In order to address this issue, two main strategies have been proposed for classical control charts: (i) fitting a model to capture the in-control dynamics of the process and subsequently monitoring its residuals (i.e., the difference between observed values and those predicted by the model) and (ii) modifying the control charts to explicitly account for autocorrelation.

The primary family of models based on the first approach (i.e., monitoring of residuals) consists of ARIMA models (e.g., [92,93,94]), although other techniques, such as the Kalman Filter, have been used for more complex processes [95]. The limitations of these models have been extensively discussed [96,97]. Regarding the second strategy, various adaptations of control charts have been proposed. Examples in the case of EWMA include EWMAST [98], AEWMA [99], and Modified EWMA [100]. Other alternatives include control charts such as the ARMA Chart [101]. Most of these strategies, however, focus on relatively simple autocorrelation models (e.g., AR(1)), which are typical in many industrial processes.

Beyond classical SPC charts, which are focused on detecting shifts in central tendency measures (e.g., mean) or variability (e.g., standard deviation), Control Chart Patterns (CCPs) are designed to identify a broader range of patterns (e.g., cyclic, systematic, or mixture patterns). In the CCP area, it is common to consider the existence of autocorrelation [102]. Unlike most classical SPC charts, many PCC approaches do not remove autocorrelation. Instead, it is integrated into the models to detect changes in variability patterns [103].

Similarly, in order to diagnose an STF, which is a specific variability pattern, it is not advisable to eliminate autocorrelation. If it were modeled using one of the aforementioned classical strategies, the control chart would detect general changes in the temporal dynamics of T or RH. However, such changes are not particularly useful in the context of preventive conservation, and, furthermore, they fail to identify specific alarm signals related to STFs. In contrast, an STF is defined as a sequence of observations that, collectively and over a relatively short period, exhibit excessive increases or decreases. Therefore, in this case, autocorrelation is not a limitation but precisely the feature that enables the detection of an STF. This integration of the autocorrelation is comparable to other reported methods [68,69] for implementing control charts in climatological studies (discussed in Section 2.2).

Therefore, in this research, it was decided to establish the control limits by determining the appropriate constants so that the CHEWMA chart can detect the desired STFs. Such a parameter choice has to be decided by preventive conservation experts and the staff of cultural heritage institutions, who must consider which amount and magnitude of STFs are acceptable for the preventive conservation of artifacts according to the institution objectives.

## 3. Materials and Methods

### 3.1. Datasets

#### 3.1.1. Dataloggers at the Alava Fine Arts Museum

The Alava Fine Arts Museum, located in Vitoria-Gasteiz, Basque Country (Spain), comprises three buildings: the historic Palacio Augustin Zulueta, a three-story annex built in the 1960s, and a visitor entrance added in 2001. A detailed description of the museum and the characterization of its microclimates are provided in a previous study [22], which serves as a reference for the present research.

As part of the European research project CollectionCare, ten prototypes of a newly developed wireless datalogger were installed in the museum in July 2020. These prototypes comply with the metrological specifications and methods outlined in EN 16242:2012 [104] and EN 15758:2010 [105]. The dataloggers began recording relative humidity (RH) and temperature (T) data on 22 July 2020. The data used for this study were collected from 1 January 2021 to 1 February 2022, comprising a period of 13 months, which is sufficient for the methods applied in this research.

To maintain consistency with the nomenclature assigned in the previous study [22], the dataloggers are labeled as follows: A, d02, d04, d05, d06, d08, and d09. Dataloggers d01, d03, and d07 were excluded from the analysis due to connectivity failures that resulted in significant data loss. Sensor A was assigned a unique label not associated with a number because no prior analogous datalogger existed in the location where it was installed. According to Díaz-Arellano et al. [22], the dataloggers used in this study exhibit a missing data rate of 4.39%. Despite this, most data gaps are minor, with 52% consisting of no more than three consecutive missing values, and 74% being limited to gaps of up to 12.

The missing data are primarily attributed to connectivity issues with the collection system and are encountered simultaneously across all time series. This fact complicates effective data imputation. However, such challenges are typical in microclimate monitoring within real-world environments. Therefore, the consideration of these limitations provides a realistic scenario for testing STF detection methods.

The dataloggers recorded one observation per hour. This recording frequency is assumed throughout the study unless otherwise specified. The analysis prioritizes RH due to its critical role in preventing mechanical damage to hygroscopic materials, which are present in numerous cultural heritage collections.

#### 3.1.2. Simulated Time Series

The time series recorded by the dataloggers were used for applying STF detection methods under real microclimatic conditions. However, having a larger number of time series enables more robust conclusions by averaging the evaluation metrics across different STF detection methods. To achieve this, 70 time series were simulated based on the 7 observed series by generating 10 simulated series for each observed series applying bootstrapping within the frequency domain. This approach, rather than generating entirely new simulated series, preserves the spectral characteristics of the recorded RH series, ensuring that STF detection methods are tested under realistic conditions. Details of the simulation process are provided in Appendix B. The simulation procedure was implemented in Python 3.13.

First, the observed time series were transformed into the frequency domain using the Fast Fourier Transform (FFT). Then, for each time series, the periodic components associated with daily and seasonal cycles were preserved, and their magnitude was randomly modified within a range of ±20%. This ensures the conservation of key patterns in the evolution of RH. Subsequently, the remaining frequencies were randomly altered in their phases to introduce diversity into the autocorrelation structure. Finally, the signals were transformed back into the time domain using the Inverse Fast Fourier Transform (IFFT), resulting in the simulated series.

The validation of the simulated series was conducted through Principal Component Analysis (PCA). For each observed series, together with the set of ten corresponding simulated series, a PCA model was fitted. The block of series was considered validated if, in each model, the Hotelling’s $T^{2}$ chart based on the four principal components showed that the time series are randomly distributed (i.e., their projections in the model’s latent space do not exhibit specific patterns among themselves). In this way, the series are considered reliable if their projections in the model’s latent space are consistent with those of the observed series and do not exhibit distinct systematic behavior. Additionally, the simulated time series were also validated through visual inspection, verifying that they do not exhibit anomalous trends or atypical behaviors that would not be expected in a real RH time series.

The simulated series were sequentially labeled from one to ten, with each label preceded by the code of the datalogger from whose time series they were derived. For example, the seventh simulated series resulting from datalogger d02 was labeled as d02.7.

### 3.2. Criteria for STF Detection According to Reference Standards

The performance of CHEWMA was evaluated against existing methods in the literature for STF detection. As outlined in Section 1, these methods can be grouped into two categories based on their approach: material-specific and multi-material methods. The CHEWMA chart, inspired by the principles underlying the EN 15757:2010 standard, fits within the second category. Consequently, its performance was evaluated in relation to two methods widely referenced in preventive conservation within the same category. The first, SMA-FT (Simple Moving Average-Flexible Thresholding), is a research method inspired by the principles of the EN 15757:2010 standard and referred to by this name in this work. The second is a threshold criterion frequently discussed in the literature, exemplified by the ASHRAE guidelines, and referred to in this work as Δ-Threshold. Both the CHEWMA chart and SMA-FT will also be compared to the application of the EN 15757:2010 standard. Although this standard is not intended for real-time monitoring of RH or T, this comparison is relevant since both methods are research proposals aimed at applying the standard in real time.

The EN 15757:2010 norm provides a standardized approach for establishing RH and T limits to minimize mechanical damage caused by STFs in artifacts composed of hygroscopic materials. Its approach involves an offline analysis of the microclimatic conditions to which the artifacts have “acclimatized” in order to identify STFs that may pose a risk.

To apply this standard, a 13-month time series of RH or T records is required, depending on the purpose of the analysis to be performed. Of these 13 months, data from one month are required to calculate a centered moving average (CMA) so that seasonal variability is derived from the remaining 12 months. The CMA is computed using a 30-day window by averaging records from the preceding and following 15 days at each time point, which captures seasonal trends. Fluctuations are defined as the deviations of the recorded values from the CMA (i.e., the difference between recorded observations and the CMA).

This standard defines a safe band (SB), beyond which fluctuations are considered the most risky fluctuations and should be avoided. Regarding how to calculate this SB, the standard states that “the lower and upper limits of the target range of RH fluctuations are determined as the 7th and 93rd percentiles of the fluctuations recorded in the monitoring period, respectively”. Although it is common in the literature to interpret and apply this rule as the establishment of fixed limits, the implementation by Camuffo et al. [106] shows how these limits form a variable-width band calculated using a moving window. In the present study, these percentiles were calculated using an additional centered 30-day window, taking the 15 days before and the 15 days after.

As mentioned in Section 2.6.2, the standard establishes what we refer to as the 10% RH criterion. If this criterion is applied, the upper limit at a given instant *i* can be defined as

(13) $$UP_{i}=\max\left(\mathrm{percentile}\;\mathrm{limit},{\mathrm{CMA}}_{i}+10\%\right).$$

This standard cannot be used for real-time monitoring because the calculation of a CMA would require future records for its computation. For real-time STF detection, some studies have proposed a new method inspired by the standard that uses a simple moving average (SMA), which relies solely on past records [43]. For an adaptation as faithful as possible to the functioning of the standard, this study first used an SMA to calculate the seasonal cycle, followed by another window of the same size to compute the percentiles and establish the SB. For example, if a 30-day temporal window is used to model the seasonal cycle, an additional 30 days will be used to construct the SB. As mentioned above, this research method is referred to in this work as SMA-FT.

The standard emphasizes the use of a single 30-day window to establish the safe band of the norm (this issue is particularly discussed in [106]). This approach establishes a general protective framework suitable for the majority of European heritage institutions. However, some studies have examined the effects of applying CMA with smaller windows for specific research cases, aiming to develop new tools for environmental diagnostic purposes and detect faster STFs (e.g., [13,20]). To ensure a meaningful comparison with CHEWMA, this study also expands the SMA-FT configurations to encompass time windows ranging from 30 to 3 days, referred to as $win_{days}$. Moreover, modifying the SMA-FT configurations with different $win_{days}$ values is useful for studying the appropriate relationship between EN 15757:2010 and SMA-FT. Although it has traditionally been assumed that configuring SMA-FT with $win_{days}=30$ would make it behave analogously to the standard, this assumption has not been demonstrated.

On the other hand, ASHRAE guidelines propose another method for STF detection, identifying RH shifts that exceed ±5 or ±10 percentage points depending on the applied control class defined in the guidelines. This approach aligns with many other standards and guidelines. Although the period for these shifts is not specified, the preventive conservation literature usually considers a 24-h interval, which is the criterion adopted here. This detection method, introduced above, is denoted as Δ-Threshold with its two possible configurations, changes greater than ±5 or ±10, referred to as Δ5 and Δ10, respectively.

### 3.3. Evaluation

In order to evaluate the similarity among the various methods and to analyze their behavior, the Sørensen–Dice Index (SDI, Equation (14)) is first introduced as a basis for our proposed modification. In this index, $S_{A}$ denotes the set of signals from the application of method A and $S_{B}$ the set of signals from method B. The index can be calculated as

(14) $$Sø\mathrm{rensen}-\mathrm{Dice}\;\mathrm{Index}=\frac{2|S_{A}\cap S_{B}|}{|S_{A}\left|+\right|S_{B}|}.$$

This index provides a practical measure of concordance in STF detection by evaluating the extent to which CHEWMA identifies the same STF as the reference methods. For example, if two methods detect the same number of signals, 100 each (i.e., $|S_{A}\left|=\right|S_{B}|=100$), and 30 of these signals coincide at the same time points (i.e., $|S_{A}\cap S_{B}|=30$), the resulting Sørensen–Dice Index would be 0.3.

**Figure 5:**
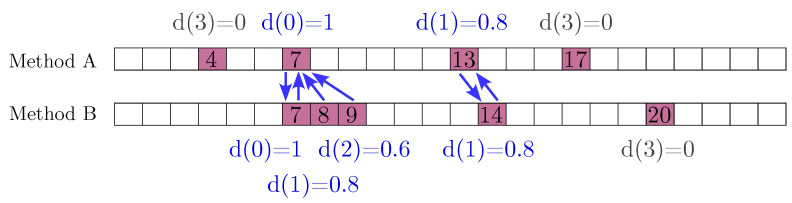
Fictitious example to illustrate the calculation of SDI* on the signals generated by methods A and B. It can be observed that, despite the apparent similarity between both methods A and B, SDI=0.22 (Equation (14)) since there is only one exact match and the two methods together generate a total of nine signals. On the other hand, as indicated by the distance calculations in the figure, SDI*=0.55 (Equation (15)), a value that may better quantify the similarity between the two series.

Although this index is intuitive, it requires exact temporal coincidence between signals to judge similarity. Since the methods being compared produce a relatively low number of signals relative to the series length (e.g., 14%) and the number of alarms generated is approximately the same but not equal, when this index is applied directly, it may underestimate the similarity between the methods, as illustrated in Figure 5. Therefore, for this study, we propose a weighted and tolerance-based modification of the SDI, referred to as SDI* (Equation (15)), which enables flexible temporal matching with weights based on proximity.

(15) $$\mathrm{SDI}*=\frac{\sum_{s_{A}\in S_{A}}w\left(d\left(s_{A},S_{B}\right)\right)+\sum_{s_{B}\in S_{B}}w\left(d\left(s_{B},S_{A}\right)\right)}{|S_{A}\left|+\right|S_{B}|}.$$

Here, d(s,S) represents the minimum temporal distance between a signal *s* in one set and any signal in the other set, formally $d\left(s,S\right)=\min_{s^{\prime }\in S}\left|t\left(s\right)-t\left(s^{\prime }\right)\right|$, where t(s) is the timestamp of signal *s*. The weight function w(d) is defined piecewise as

(16) $$w\left(d\right)=\left\{\begin{array}{cc} 1 & \mathrm{if}\;d=0,\\ 0.8 & \mathrm{if}\;d=1,\\ 0.6 & \mathrm{if}\;d=2,\\ 0 & \mathrm{if}\;d>2. \end{array}\right.$$

In simple terms, SDI* (Equation (15)) first attempts to match signals exactly, just as SDI (Equation (13)) does (i.e., signals generated at the same time). In this case, it assigns the highest possible score, ensuring that w(0)=1. If an exact match is not found but the closest signal is at a distance of one, a lower score is assigned such that w(1)=0.8. The same applies to a distance of two, with a lower score, where w(2)=0.6. In any other case, no match is made. A specific example of its application can be seen in Figure 5.

As with the original SDI, the weighted version with tolerance (SDI*) ranges from zero to one (SDI*∈[0,1]), representing no similarity and complete similarity, respectively, making it easy to interpret. This adjustment ensures that near-matches contribute proportionally to the similarity score, mitigating underestimation issues caused by the strict temporal matching requirement of the original SDI.

The coding for the STF detection methods and their evaluation was implemented in the statistical software R 4.3.1.

## 4. Results and Discussion

### 4.1. Simulated Time Series Validation

To validate the simulated series, as outlined in Section 3.1.2, seven PCA models were constructed, each comprising one recorded series and the ten series generated from it (e.g., the time series from datalogger d06 was included along with the simulated series d06.1 to d06.10). Each model was fitted considering four principal components because, in general, the explained variability began to decrease significantly beyond the fourth component. Moreover, with these four components, more than 50% of the data variability is explained. Specifically, the variability explained by each model is as follows: A, 55.22%; d02, 53.05%; d04, 51.09%; d05, 56.09%; d06, 53.31%; d08, 56.66%; and d09, 51.39%.

**Figure 6:**
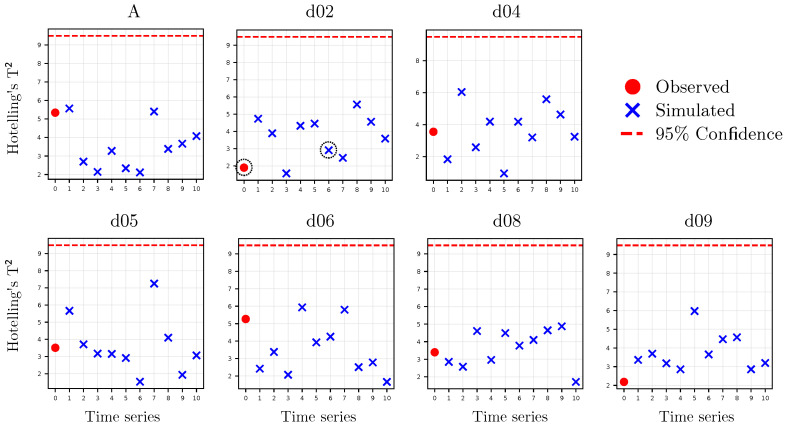
Hotelling’s $T^{2}$ statistic plots for each PCA model fitted with four components. In each plot, the statistic for the observed series (e.g., d06) is displayed first, followed by the ten simulated series (e.g., d06.1 to d06.10). The time series marked with a circle in d02 correspond to the series in Figure 7. All Hotelling’s $T^{2}$ statistics remain below the 95% confidence limit and exhibit no specific patterns.

The Hotelling’s $T^{2}$ statistic plots (Figure 6) show that the observed and simulated series are distributed in the same latent space without presenting anomalous patterns and remaining within the established 95% confidence interval.

**Figure 7:**
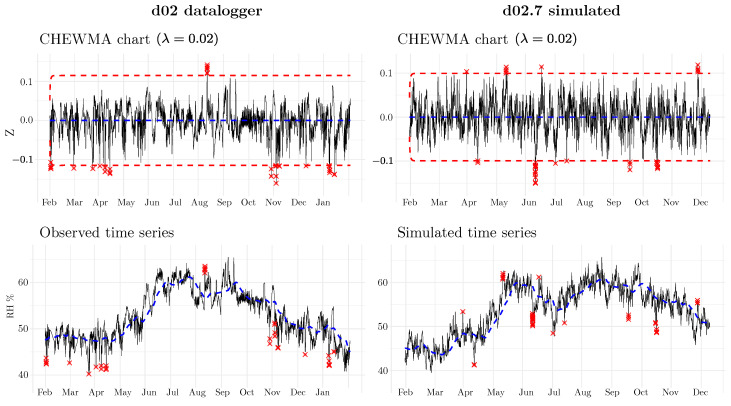
Examples of the application of CHEWMA chart (λ=0.02) on an observed time series (d02, left) and a simulated series derived from it (d02.7, right). The upper panels show the control charts monitoring the *Z* statistic (in black), with the mean of the statistic equal to zero ($\mu_{Z}=0$, in blue), the upper and lower limits (in red), and the signals crossing the limits (in red). The lower panels display the alarm signals (in red) superimposed on the observed time series (*X*, in black) and the 14-day moving average (${\hat{\mu }}_{X_{t}^{24h}}$, in blue). The control limits were set to detect 0.5% of the observations as alarms. This percentage was chosen to highlight a reduced number of STFs, attempting to simulate real-world usage.

On the other hand, the appearance of the simulated series shows a realistic temporal dynamic. Figure 7 shows the application of the CHEWMA chart on the time series of datalogger d02 and a simulated series derived from it, d02.7. Although both series share similarities because the simulated series was generated by maintaining the daily and seasonal cycle, they also show clearly differentiated patterns. Such patterns, introduced randomly, are distributed throughout the entire time series, realistically reproducing the natural evolution of RH over time. The similarity between both series is evident in that they generate the same percentage of signals (0.5%) with similar limit sizes (L=0.51 for d02, and L=0.44 for d02.7 with λ=0.02). At the same time, the differences are apparent in the pattern of signal distribution.

### 4.2. STF Detection Based on Fluctuation Speed: Sensitivity of the Parameter λ

To examine the sensitivity of the CHEWMA chart to changes in the parameter λ, all the control chart configurations in this subsection were fixed considering β=0. In addition, unless otherwise specified, the 10% criterion will not be applied to the results in order to accurately compare the behavior of the different procedures, avoiding the use of a common criterion that would homogenize them and hinder the analysis of differences.

**Figure 8:**
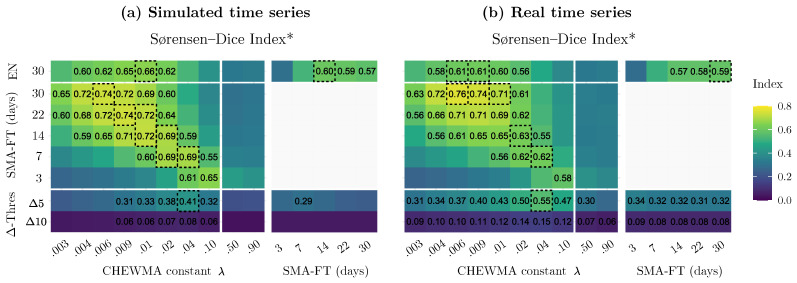
Similarity of the methods with respect to the CHEWMA chart and SMA-FT using simulated and real data, calculated using SDI* (Equation (15)). Each cell value represents the average index calculated over the set of simulated series in (**a**) and real time series in (**b**). The cells outlined with dashed lines compare matching configurations with highest similarity. For a better comparison, CHEWMA and SMA-FT were adjusted to identify ∼14% of the observations as signals. To improve clarity, values below 0.55 are omitted in the rows of SMA-FT and EN 15757:2010 as well as below 0.28 and 0.06 in the rows of Δ5 and Δ10, respectively.

Figure 8 compares the similarity between the CHEWMA chart and SMA-FT, both with each other and with EN 15757:2010, Δ5, and Δ10, applied to both real and simulated time series. Although the results for both sets of series generally exhibit similar patterns, the application to the set of simulated series appears to be more consistent. For example, it is more evident how CHEWMA chart applications with a larger λ can be matched with SMA-FT with a smaller $win_{days}$ in a clear diagonal pattern. Therefore, while the application to real series is valuable for verifying that the results from both sets are consistent with each other, this study will use the results from the simulated dataset as a reference as they exhibit greater robustness, which is an expected outcome given that the simulated dataset contains ten times more time series.

**Figure 9:**
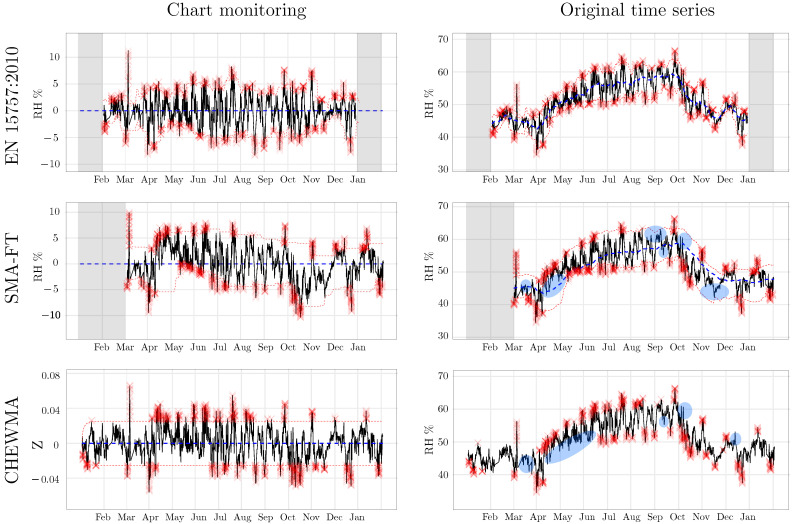
Comparison between the application of the EN 15757:2010 standard, SMA-FT ($win_{days}=30$), and CHEWMA (λ=0.06) on the RH time series recorded by datalogger d08. This time series was selected to highlight the differences between the three methods, which are less noticeable when applied to other time series. For a better comparison, CHEWMA and SMA-FT were adjusted to identify approximately 14% of the observations as signals. Charts in the left column depict the application of each method’s procedures. For EN 15757:2010 and SMA-FT, this includes fluctuations and the SB derived from percentiles. For the CHEWMA chart, it consists of monitoring the *Z* statistic and its limits. Charts in the right column represent the application of the generated signals to the original time series. The blue areas indicate discrepancies with the application of the EN 15757:2010 standard. The gray areas indicate the periods discarded by EN 15757:2010 and SMA-FT, which are required for the calculation of moving windows.

The most noticeable observation provided by the similarity analysis using SDI* is the strong resemblance between the CHEWMA chart and SMA-FT, which exceeds 0.70 for common configurations with high $win_{days}$ values. This similarity is also apparent when closely examining the signals generated by both methods on the time series recorded by datalogger d08 (Figure 9). Moreover, the results comparing the CHEWMA chart and the EN 15757:2010 standard indicate notable similarity between them (SDI* = 0.66), which is also reflected in the specific application to the time series from datalogger d08. This similarity is greater than that observed between SMA-FT and EN 15757:2010 (SDI* = 0.60) for the SMA-FT configuration with $win_{days}=14$, which is the most comparable to the European standard among those studied.

To assess the statistical significance of the greater similarity between the CHEWMA chart and EN 15757:2010, the difference in similarity was computed for each application of the CHEWMA chart and SMA-FT across the 70 time series. All the differences were positive (i.e., CHEWMA was more similar to EN 15757:2010 in every time series). These differences, which approximately follow a normal distribution, showed in a hypothesis test that their mean (0.063) is significantly greater than 0, specifically demonstrating a *p*-value $<2.2\times 10^{-16}$, leading to the conclusion that, for the studied sample, the application of the CHEWMA chart is more similar to the results of the EN 15757:2010 standard.

It is remarkable that the SMA-FT configuration most similar to EN 15757:2010 is not the one with $win_{days}=30$ (which achieves an SDI* = 0.57) but rather the one with $win_{days}=14$ (which achieves an SDI* = 0.60). These results are consistent with other tested configurations that are approximately half of $win_{days}=30$. Specifically, $win_{days}$ values of 12, 13, and 15 yield the same SDI* = 0.60. This finding is also consistent with the comparison between the application of EN 15757:2010 and SMA-FT ($win_{days}=30$) (Figure 9). It can be observed that some periods in which SMA-FT does not generate signals for the same observations as EN 15757:2010 are due to an excessively wide SB. For example, this situation occurs in April and November because, when the same window is applied using CMA and then using SMA, the latter tends to overrepresent distant past observations, considering a different seasonal cycle than EN 15757:2010. The same phenomenon can also be observed in the rigidity of the SB generated by SMA-FT, which changes abruptly throughout the months, while the SB calculated by EN 15757:2010 adapts better to fluctuations. From these results, we conclude that the appropriate SMA-FT configuration to approximate the use of EN 15757:2010 corresponds to intermediate $win_{days}$ values (e.g., $win_{days}=14$) rather than 30, as has been reported by previous studies.

For the dataset studied here, the similarity between the CHEWMA chart and the Δ-Threshold criteria is low (Figure 8). Indeed, the similarity between both methods is highly dependent on the dataset used. For example, when applying them to time series with greater intraday variability, the number of signals detected by Δ5 and Δ10 would increase, generating signals that would also generally be identified by the CHEWMA chart. It is interesting to note that the CHEWMA chart exhibits greater similarity to the Δ-Threshold criteria than SMA-FT. This result suggests that the CHEWMA chart has a greater tendency to detect abrupt changes occurring in short temporal windows (e.g., 24 h) while also maintaining the capacity to detect changes occurring over longer periods.

Figure 9 reveals the different distributions of signal generation throughout the year among the CHEWMA chart, SMA-FT, and EN 15757. It can be observed that EN 15757:2010 tends to generate approximately the same number of signals throughout the year. Additionally, the signals are distributed in similar quantities both above and below the seasonal cycle. For example, unlike EN 15757:2010, during April, neither the CHEWMA chart nor SMA-FT produced signals below the seasonal cycle. This period extended into the following month in the case of the CHEWMA chart.

**Figure 10:**
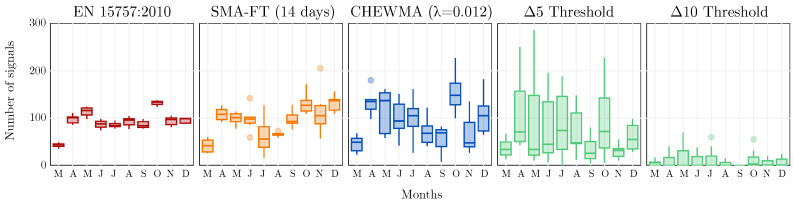
Monthly distribution of the number of signals detected by each method evaluated in this study, applied to each of the real time series. The comparison is conducted between methods with similar configurations: EN 15757:2010, SMA-FT ($win_{days}=14$), and CHEWMA (λ=0.012). For better comparison, CHEWMA and SMA-FT were adjusted to identify approximately 14% of the observations as signals. Although all time series were generated from dataloggers within the same museum and therefore exhibit very similar seasonal patterns, each of them presents unique characteristics. Specifically, two of them, d04 and d06, are located in a different building with distinct microclimatic conditions compared to the rest [22].

This difference has been further analyzed by examining the distribution of signals generated by each method for each month throughout the year (Figure 10). In this comparison, EN 15757:2010 exhibits more homogeneous behavior than the other methods, detecting approximately the same number of signals regardless of the variability in the month or the specific time series to which it is applied. In contrast, SMA-FT and, especially, the CHEWMA chart detect more or fewer signals depending on the variability in the time series. However, in certain identified patterns, the standard and the two methods show significant agreement. For example, all three procedures generate few signals in March across all the time series, and there is a general decline from April to July. Although with varying intensity, a pattern of a high number of signals in October, a decrease in November, and another increase in December is also observed. However, the distributions of Δ5 and Δ10 are very different and strictly dependent on the specific time series to which they are applied. Notably, since both criteria do not account for seasonal changes but are restricted to changes within a 24-h range, they tend to exhibit a certain degree of homogeneity throughout the year.

This study has investigated the relationship between EN 15757:2010 and SMA-FT through their window sizes and the width of the SB (SBW), which has turned out to be a key factor in linking both methods. Camuffo et al. [106] showed that the relationship between these two factors, $win_{days}$ and SBW, follows a logarithmic relationship. In their study, they examined this behavior by fitting a simple linear regression between the different $win_{days}$ values studied and the SBW mean they produced, denoted as $\bar{SBW}$. This relationship is expressed as

(17) $$\bar{SBW}=b_{0}+b_{1}\times \ln\left(win_{days}\right).$$

**Figure 11:**
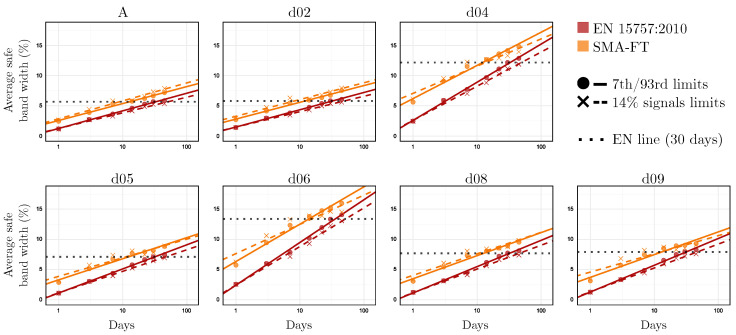
Analysis of the logarithmic relationship between $win_{days}$ and $\bar{SBW}$ for EN 15757:2010 (red) and SMA-FT (orange). Temporal windows are applied within the range $win_{days}=\left(1,3,7,14,22,30,45\right)$. This relationship is analyzed in two different ways to construct the SB. First, without altering the percentiles used to determine the limits, keeping them at the 7th and 93rd percentiles, as conducted in [106], which can be observed in the circles and the solid line. Second, by adjusting the percentiles to identify approximately 14% of the observations as signals, which slightly differs from setting the limits at the 7th and 93 rd percentiles when applying a moving temporal window. The horizontal dashed lines help to visualize the relationship between EN 15757:2010 adjusted with $win_{days}=30$ and SMA-FT adjusted with $win_{days}=14$.

To validate and further explore this relationship, the same analysis is conducted here for EN 15757:2010, and, as a novel contribution, it is also applied to SMA-FT to compare both methods. As shown in Figure 11, SMA-FT maintains the same logarithmic relationship between $win_{days}$ and $\bar{SBW}$. Therefore, this relationship exists not only when the SB is calculated using a CMA but also when an SMA is applied. Another key finding is that, for the same $win_{days}$, SMA-FT systematically generates wider SBs. This is consistent with the results discussed above. In particular, it can be observed that, in general, EN 15757:2010, as defined by the standard (i.e., with $win_{days}=30$), generates an $\bar{SBW}$ approximately equal to that of SMA-FT with $win_{days}=14$. Therefore, if SMA-FT is used as an approach to applying the principles of EN 15757:2010 in real time, it is advisable to conduct this preliminary analysis to determine the appropriate $win_{days}$ corresponding to the standard’s application for greater accuracy. However, as a general rule and as discussed above, $win_{days}=14$ can be used as a reasonable approximation.

It is also interesting to note that the time series recorded by dataloggers d04 and d06, which exhibit greater variability throughout the year [22], also generate two lines with more pronounced slopes (i.e., higher $b_{1}$ values), distinguishing them from the rest of the dataloggers located in a different building of the museum.

When EN 15757:2010 and SMA-FT are not applied with the 7th and 93rd percentiles but are instead adjusted to generate an approximate number of signals (in this case, ∼14%), the relationship between the standard and SMA-FT remains, meaning that SMA-FT continues to have a systematically greater $\bar{SBW}$ in a similar proportion.

When comparing the logarithmic relationship established between the applications of EN 15757:2010 using fixed percentiles and adjusted percentiles, all the models show a very similar $R^{2}$, around 0.99. However, the logarithmic relationship between both adjustments differs in the case of SMA-FT. While models adjusted using fixed percentiles maintain an $R^{2}$ within the range [0.95, 0.99], the linear regression applied to the adjusted models designed to generate an approximate number of alarms yields an $R^{2}$ within the range [0.81, 0.97]. This difference arises because, while EN 15757:2010 does generate an approximate alarm percentage of 14% when the percentiles are set at the 7th and 93rd percentiles, SMA-FT generates a different number of alarms when the SB is calculated using the same percentiles. Therefore, the true logarithmic relationship is between $win_{days}$ and $\bar{SBW}$, whereas the relationship between $win_{days}$ and the percentage of alarms generated is less direct and merely approximates a logarithmic pattern as a by-product of the former. These results also indicate that SMA-FT is a less predictable method than the EN 15757:2010 standard as the percentiles diverge further from the expected percentage of generated signals.

**Figure 12:**
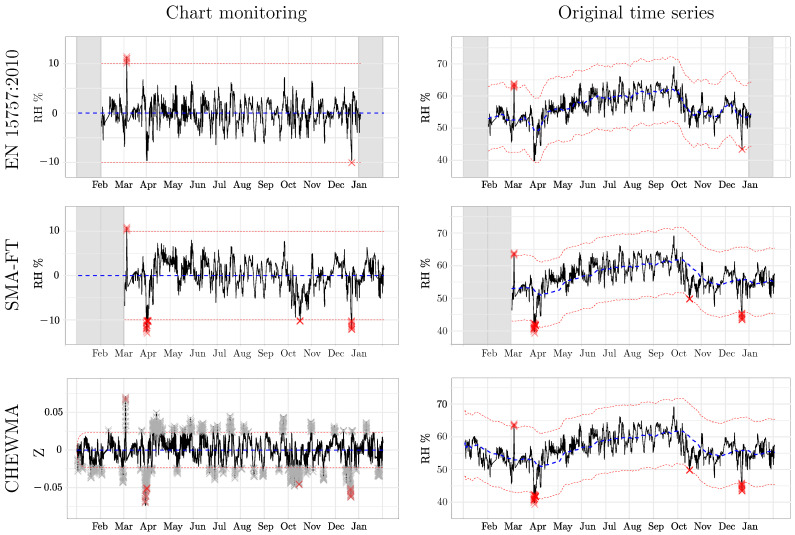
Comparison between the application of the EN 15757:2010 standard, SMA-FT ($win_{days}\;=\;30$), and CHEWMA (λ=0.06) applying the 10% criterion to the RH time series recorded by datalogger d08. As in Figure 9, for better comparison, CHEWMA and SMA-FT were adjusted to identify approximately 14% of the observations as signals. Charts in the left column depict the application of each method’s procedures. For EN 15757:2010 and SMA-FT, this includes fluctuations and the SB derived from percentiles. For the CHEWMA chart, it consists of monitoring the *z* statistic and its limits. Charts in the right column represent the application of the generated signals to the original time series. The CHEWMA chart displays signals generated by the chart limits, shown in gray, that fall below the 10% threshold. The gray areas indicate the periods discarded by EN 15757:2010 and SMA-FT, which are required for the calculation of moving windows.

The EN 15757:2010 standard, SMA-FT, and the CHEWMA chart exhibit even greater similarities when the 10% criterion is applied (i.e., if the artifact is considered to be properly preserved, an STF that departs by less than 10% may be deemed acceptable). In Figure 12, it can be observed that, when applying the mentioned criterion to the time series from datalogger d08, the EN 15757:2010 standard generates two signals: one in the first days of March and another at the end of December. These signals are also identified by the CHEWMA chart and SMA-FT, as well as additional signals in early April, which EN 15757:2010 is also close to detecting, and others in mid-October. However, it should be noted that, in the comparisons shown in Figure 9 and Figure 12, the CHEWMA chart and SMA-FT configurations used are not the closest to EN 15757:2010 based on the similarity analysis (Figure 8) but rather those corresponding to the application of $win_{days}=30$.

As can be observed in the applications to d08 (Figure 9 and Figure 12), the ability of the CHEWMA chart to operate immediately, without the need to wait for a temporal window to be calculated, is a significant advantage over SMA-FT. Regarding interpretation, having an SPC chart with fixed limits (except during the first days after its implementation, when the limits progressively increase) facilitates the analysis of fluctuation trends (Figure 9). However, when applying the CHEWMA chart with the 10% criterion, it is necessary to consider two pairs of limits simultaneously, which may be confusing (Figure 12).

For specific applications outside the general framework established by the EN 15757:2010 standard, the CHEWMA chart can be configured for the detection of rapid STFs occurring within minutes or a few hours by using large lambda values (approximately λ=(0.2,1] for hourly sampling frequency). This could be useful for specific cases involving fragile artifacts with very high moisture absorption rates and can be configured using the appropriate *L* constant to generate the percentage of alarms considered to be suitable by the user for the needs of the heritage institution. For such applications, CHEWMA proves to be more flexible than SMA-FT. For instance, if SMA-FT were to be used to detect very rapid STFs, it would require a very small temporal window (e.g., one day) and extreme percentiles (e.g., 3 and 97). In the common case of monitoring RH or T at an hourly frequency, SMA-FT would tend to detect one signal per day. Although this fluctuation may be large compared to the last 24 h, it is not necessarily particularly high; therefore, the user might interpret such a signal as a false positive. This suggests that, while the moving window approach is appropriate for modeling the seasonal cycle and identifying the STFs that should be avoided based on it, it does not appear to be equally effective when adapted exclusively for the detection of abrupt changes irrespective of that cycle as Δ-Threshold does.

### 4.3. STF Detection at Extreme Values: Sensitivity of the Parameter β

**Figure 13:**
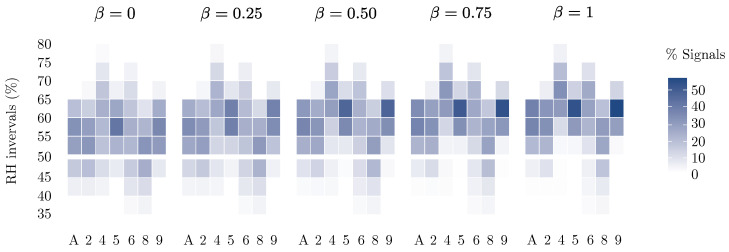
Percentage of RH signals detected by the CHEWMA chart (λ=0.1, p=50%) as a function of changes in β. The chart was applied to the simulated time series with β=0,0.25,0.5,0.75,1. The amount of resulting signals has been averaged per datalogger. The chart limits were adjusted to detect 5% of the observations as signals. As β increases, it can be appreciated that a higher proportion of signals is distributed towards extreme values. In this case, the distribution skews more towards high extremes (near 100% RH) than towards low extremes (near 0% RH) due to the greater number of fluctuations occurring near 100% RH in this time series. For clarity, the time series from each datalogger is labeled with its respective number (e.g., d02.1, …, d02.10 are averaged and correspond to label 2).

While λ adjusts the chart’s overall sensitivity to the speed of temporal shifts in the time series, β adjusts the relative importance of observations near the extremes of the RH range of measurement. This enables targeted detection of STFs that occur close to critical RH values. As shown in Figure 13, when β=0, the chart performs like a standard EWMA chart, with fluctuations weighted uniformly and distributed around the annual mean of the time series. As the weighting towards extreme values increases (i.e., as β increases), the distribution of alarms shifts from the central range to values closer to 100% or 0% RH.

This performance is particularly useful in conservation contexts where RH fluctuations near critical thresholds imply significant risks to the integrity of cultural heritage objects. For example, according to the guidelines of [85], RH fluctuations below 35% should be avoided to prevent mechanical damage to materials such as teeth, bone, and shell. In a hypothetical scenario involving a set of rooms containing artifacts of this type, the CHEWMA chart could be configured with a reference point p=55 and a positive β value (β>0), prioritizing the detection of critical RH drops.

An additional potential application of the CHEWMA chart is to address the challenges associated with the movement of artifacts between different environments. Artifacts may be relocated for various reasons, such as exhibitions, conservation treatments, or institutional loan agreements. When artifacts are moved from the microclimate to which they have been historically acclimatized, they are exposed to environmental conditions that might differ significantly from those they were previously adapted to. This issue represents a significant hazard for the mechanical properties of the artifact (for a practical discussion in this regard, see [107]).

The weighting mechanism, and particularly the contextual reference provided by the parameter *p*, enables the CHEWMA chart to retain this historical microclimate as a baseline for STF detection. Once the artifact is moved to a different microclimate, the reference point *p* can be set to the acclimatized annual mean while retaining the values of the other constants (such as λ and β), penalizing deviations from the original microclimate. These parameters could serve as the basis for defining climate control agreements in these contracts, ensuring that the artifacts remain within safe environmental conditions. This approach offers a balanced solution between establishing shared thermohygrometric conditions between both institutions and adjusting these restrictions to preventive conservation criteria based on the historical acclimatization of artifacts.

The flexibility of the CHEWMA chart is advantageous for adapting to specific situations but may also present challenges if the appropriate configuration is unclear. This additional complexity in selecting suitable constants can be addressed through a combination of understanding the institution’s conditions and needs along with iterative testing on historical data to identify parameter values as part of staff training. For example, setting restrictive limits would lead to generating a higher percentage of alarm signals, but it could be advantageous if the detection system is directly integrated with automated HVAC controls. However, this approach might be less practical if each signal requires being diagnosed manually by the staff. Similarly, configuring the CHEWMA chart to detect time-prolonged STFs (e.g., λ < 0.05) could be particularly beneficial for collections with artifacts composed of materials that respond slowly to RH fluctuations. In the case of artifacts that react quickly to thermohygrometric changes, a configuration with higher λ values would be more appropriate.

## 5. Conclusions

The CHEWMA chart has been introduced as a method for monitoring RH and T, aiming to assist in controlling microclimates for preventive conservation of artifacts in museums and other cultural heritage institutions. As far as we know, this research represents the first application of statistical process control techniques in the field of preventive conservation. The applications of SPC charts to non-industrial fields, as discussed in this study, along with the results, highlight the potential of this approach.

The CHEWMA chart has been compared to two widely used methods in the preventive conservation literature for detecting STFs: SMA-FT and Δ-Threshold. In order to broaden the investigation, the EN15757:2010 standard was included in the comparison even though it is not intended for real-time monitoring. When the chart is solely intended to detect STFs regardless of whether they occur at extreme values, no weighting should be applied (i.e., β=0). This control version of the chart can be considered simpler than SMA-FT as it does not require time windows for initialization, is arithmetically straightforward, and can be operational from the very beginning of microclimate monitoring. This CHEWMA chart configuration has been shown to be significantly closer to the application of the EN 15757:2010 standard than SMA-FT. Moreover, this supports the conclusion that the CHEWMA chart basically offers the same advantages as EN 15757:2010 for real-time monitoring regarding mechanical damage prevention and energy savings. Nevertheless, as emphasized throughout the paper, neither the CHEWMA chart nor SMA-FT should be considered as alternative procedures to the EN15757:2010 standard; rather, they are adaptations for real-time STF detection inspired by the fundamental concepts of ’historic climate’ and ’acclimatization’ as established by the standard.

The relationship between EN 15757:2010 and its immediate adaptation for real-time monitoring, SMA-FT, has been studied. The results reveal that this adaptation should consider a temporal window of approximately 14 days, which is significantly shorter than the 30-day window typically assumed in the literature.

Beyond this, the CHEWMA chart offers significant flexibility in its configuration, being capable of detecting STFs across a wide temporal range, from shifts occurring within minutes or hours to those developing over days. It also enables users to define what constitutes an extreme STF through the constant *p*, and to adjust the weighting parameter β to focus detection on critical conditions that could compromise the mechanical properties of artifacts that are more vulnerable to shifts at extreme RH or T values. This flexibility would enable institutions to adapt STF detection and control to their specific conservation needs. However, this flexibility introduces a layer of complexity in selecting and fine-tuning the constants, requiring iterative testing and careful consideration of the institutional conditions to optimize implementation. Nevertheless, it offers a valuable response to a meaningful need highlighted in the preventive conservation literature: the sensitivity of many materials to extreme conditions. In such scenarios, the proposed methodology provides a novel tool based on statistical principles for incorporating this risk into the detection methods framework.

## 6. Future Work

The results of the study and the hypotheses presented reveal various possibilities for future work.

Although the conclusions of this research have been drawn after discussing the results from different perspectives, they are based on a case study involving two microclimates. Future research on new case studies would further enrich the conclusions obtained. Furthermore, another area to investigate is the extent to which CHEWMA’s weighting mechanism may be critical for materials that are sensitive to extreme values, as well as its potential utility in maintaining reasonably safe microclimatic conditions while artifacts are relocated.

In this research, the analyses were conducted on time series recorded at an hourly frequency, which is the most common in microclimatic monitoring. However, another interesting aspect to investigate is how different sampling frequencies might affect the anticipation results between methods.

Moreover, the application of SPC charts to preventive conservation opens up numerous possibilities for further development and new applications. For example, based on the present work, well-known control charts such as the CUSUM chart can be adapted. Moreover, since the CHEWMA chart is univariate, future work should explore its multivariate extension. Such an adaptation would enable simultaneous monitoring of multiple dataloggers, facilitating comprehensive oversight of cultural heritage institution conditions and enhancing decision-making in response to undesirable STFs. Such developments would bridge the gap between advanced statistical tools and their practical implementation in preventive conservation, offering new ways to protect valuable artifacts under complex environmental dynamics.

## Figures and Tables

**Table 1:** Summary of statistics for CHEWMA approaches.

	Non-Weighted CHEWMA (β=0)	Weighted CHEWMA Non-(β>0)
Required estimations	$\sigma_{\Delta X}$	$\sigma_{\Delta X},\mu_{X_{t}^{24}},\sigma_{X_{t}^{24}},{\hat{\mu }}_{X^{\mathrm{annual}}}$
Monitored statistic	$Z_{t}=\lambda \Delta X_{t}+\left(1-\lambda \right)Z_{t-1}$	$Z_{t}=\lambda W_{t}+\left(1-\lambda \right)Z_{t-1}$

## Data Availability

The simulated data are provided in the Supplementary Materials of the article. The R code used in this study is publicly available at [108].

## Supplementary Materials

The following supporting information can be downloaded at: https://www.mdpi.com/article/10.3390/s25041242/s1, Table S1: Simulated Time Series.

## Abbreviations

The following abbreviations are used in this manuscript:

STF	Short-Term Fluctuation
RH	Relative Humidity
T	Temperature
HVAC	Heating, Ventilation, and Air Conditioning
SPC	Statistical Process Control
EWMA	Exponentially Weighted Moving Average
CHEWMA	Cultural Heritage EWMA
CUSUM	Cumulative Sum Control
CMA	Centered Moving Average
SMA	Simple Moving Average
SMA-FT	Simple Moving Average-Flexible Thresholding
SB	Safe Band
SBW	Safe Band Width

## Appendix A. Mathematical Calculations for Expectation and Variance of W Statistic

Renaming the variables and adjusting the terms, Formula (6) can be simplified as

(A1) $$W=A-\beta +\frac{\beta }{p}B.$$

Assuming *A* and *B* as normal and independent variables, the average of *W* (mathematical expectation) can be calculated as

(A2) $$\begin{array}{cc} E[W] & =E\left[\left(A-\beta \right)+\frac{\beta }{p}B\right]\\ \; & =E\left[A-\beta \right]+E\left[\frac{\beta }{p}B\right]\\ \; & =\left(E\left[A\right]-\beta \right)+\frac{\beta }{p}E\left[B\right]\\ \; & =\left(\mu_{A}-\beta \right)+\frac{\beta }{p}\mu_{B} \end{array}$$

Given that *A* represents variable of increments and decrements within an annual cycle, a null average can be considered, $\mu_{A}=0$. Therefore, the final expression for the mean of *W* becomes

(A3) $$\begin{array}{cc} E[W] & =-\beta +\frac{\beta }{p}\mu_{B}. \end{array}$$

Similarly, the variance of *W* can be calculated as

(A4) $$\begin{array}{cc} \mathrm{Var}[W] & =\mathrm{Var}\left[\left(A-\beta \right)+\frac{\beta }{p}B\right]\\ \; & =\mathrm{Var}\left[A-\beta \right]+\mathrm{Var}\left[\frac{\beta }{p}B\right]\\ \; & =\mathrm{Var}\left[A\right]+{\left(\frac{\beta }{p}\right)}^{2}\mathrm{Var}\left[B\right]\\ \; & =\sigma_{A}^{2}+{\left(\frac{\beta }{p}\right)}^{2}\sigma_{B}^{2} \end{array}$$

## Appendix B. Time Series Simulation Algorithm in the Frequency Domain

**Algorithm A1** Frequency-Domain Bootstrapping for Time Series Simulation
**Require:** • Observed time series x(t)
• Number of simulations *N*
• Amplitude variation range ±α (e.g., α=0.2 for ±20%)
• Frequencies of interest: daily ($f_{\mathrm{daily}}$) and seasonal ($f_{\mathrm{seasonal}}$)
**Ensure:** • Set of simulated time series $\left\{x_{\mathrm{sim}}^{\left(1\right)}\left(t\right),\ldots ,x_{\mathrm{sim}}^{\left(N\right)}\left(t\right)\right\}$ 1: **for** each simulation i=1 **to** *N* **do** 2: **Fourier Transform:** X(f)←FFT(x(t)) 3: **Initialize Synthetic Fourier Spectrum:** $X_{\mathrm{sim}}\left(f\right)\leftarrow 0$ 4: **Preserve and Randomly Scale Daily and Seasonal Components:** 5: **for** each frequency $f_{p}\in \left\{f_{\mathrm{daily}},f_{\mathrm{seasonal}}\right\}$ **do** 6: $A_{p}^{\prime }\leftarrow A_{p}\cdot \left(1+\delta \right)$ δ∼U(−α,α) 7: $X_{\mathrm{sim}}\left(f_{p}\right)\leftarrow A_{p}^{\prime }\cdot e^{j\phi_{p}}$ 8: **if** $f_{p}\neq 0$ **then** 9: $X_{\mathrm{sim}}\left(-f_{p}\right)\leftarrow A_{p}^{\prime }\cdot e^{-j\phi_{p}}$ 10: **end if** 11: **end for** 12: **Randomize Phases of Other Frequencies:** 13: **for** each frequency $f\notin \{f_{\mathrm{daily}},f_{\mathrm{seasonal}}\}$ **do** 14: $\phi_{\mathrm{rand}}\leftarrow U\left(-\pi ,\pi \right)$ 15: A←|X(f)| 16: $X_{\mathrm{sim}}\left(f\right)\leftarrow A\cdot e^{j\phi_{\mathrm{rand}}}$ 17: $X_{\mathrm{sim}}\left(-f\right)\leftarrow A\cdot e^{-j\phi_{\mathrm{rand}}}$ 18: **end for** 19: **Handle DC and Nyquist Components:** 20: $X_{\mathrm{sim}}\left(0\right)\leftarrow X\left(0\right)$ {DC component remains unchanged} 21: **if** number of points *n* is even **then** 22: $X_{\mathrm{sim}}\left(f_{\mathrm{Nyquist}}\right)\leftarrow X\left(f_{\mathrm{Nyquist}}\right)$ 23: **end if** 24: **Inverse Fourier Transform:** $x_{\mathrm{sim}}^{\left(i\right)}\left(t\right)\leftarrow \mathrm{IFFT}\left(X_{\mathrm{sim}}\left(f\right)\right)$ 25: **end for** 26: **return**$\left\{x_{\mathrm{sim}}^{\left(1\right)}\left(t\right),\ldots ,x_{\mathrm{sim}}^{\left(N\right)}\left(t\right)\right\}$
